# Supplementary material for: A network dynamical simulation model for the study of antibiotic resistance in microbial communities
Source: Gut Microbes. 2026 Sep 19;18(1):2734703. doi: 10.1080/19490976.2026.2734703 (PMC13596919; doi:10.1080/19490976.2026.2734703)
Supplement: Supplementary file 1 [file KGMI_A_2734703_SM9053.zip › supplementary_file_1/Figures/ba_pct_delta_n_edges_n100.pdf]

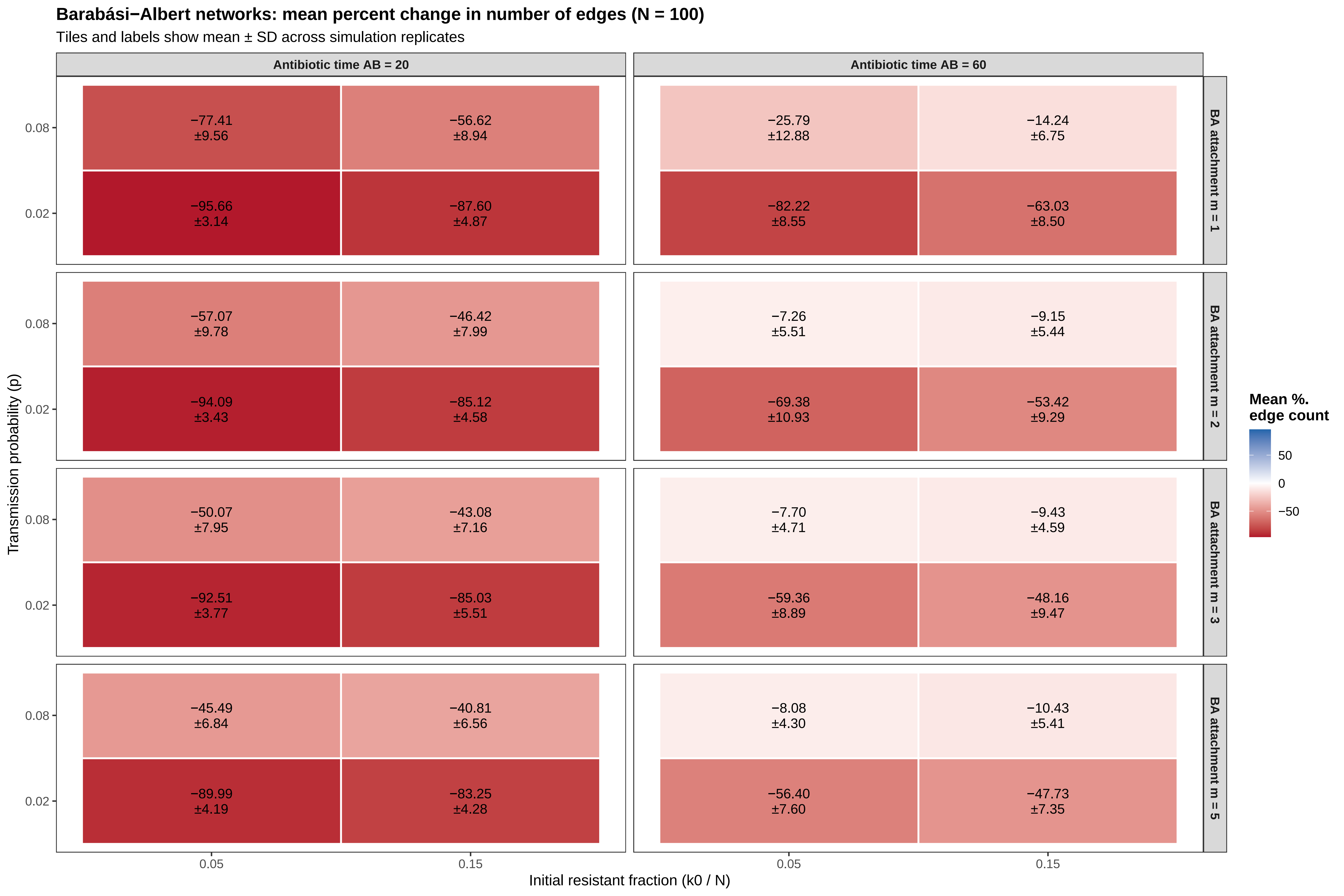

Barabási−Albert networks: mean percent change in number of edges (N = 100)
Tiles and labels show mean ± SD across simulation replicates
Antibiotic time AB = 20
Antibiotic time AB = 60
BA attachment m = 1
BA attachment m = 2
BA attachment m = 3
BA attachment m = 5
−77.41 ±9.56
−56.62 ±8.94
−25.79 ±12.88
−14.24 ±6.75
−95.66 ±3.14
−87.60 ±4.87
−82.22 ±8.55
−63.03 ±8.50
−57.07 ±9.78
−46.42 ±7.99
−7.26 ±5.51
−9.15 ±5.44
−94.09 ±3.43
−85.12 ±4.58
−69.38 ±10.93
−53.42 ±9.29
−50.07 ±7.95
−43.08 ±7.16
−7.70 ±4.71
−9.43 ±4.59
−92.51 ±3.77
−85.03 ±5.51
−59.36 ±8.89
−48.16 ±9.47
−45.49 ±6.84
−40.81 ±6.56
−8.08 ±4.30
−10.43 ±5.41
−89.99 ±4.19
−83.25 ±4.28
−56.40 ±7.60
−47.73 ±7.35
Transmission probability (p)
0.08
0.02
Initial resistant fraction (k0 / N)
0.05
0.15
Mean %. edge count
50
0
−50
